# Non-invasive imaging of allogeneic transplanted skin graft by ^131^I-anti-TLR5 mAb

**DOI:** 10.1111/jcmm.12423

**Published:** 2014-10-06

**Authors:** Hukui Sun, Guangjie Yang, Ting Liang, Chao Zhang, Jing Song, Jiankui Han, Guihua Hou

**Affiliations:** Key Laboratory for Experimental Teratology of the Ministry of Education and Institute of Experimental Nuclear Medicine, School of Medicine, Shandong UniversityJi'nan, Shandong, China

**Keywords:** ^131^I-anti-TLR5 mAb, ^18^F-FDG, allo-treated, rapamycin, phosphor-autoradiography

## Abstract

Although ^18^F-fluorodeoxyglucose (^18^F-FDG) uptake can be used for the non-invasive detection and monitoring of allograft rejection by activated leucocytes, this non-specific accumulation is easily impaired by immunosuppressants. Our aim was to evaluate a ^131^I-radiolabelled anti-Toll-like receptor 5 (TLR5) mAb for non-invasive *in vivo* graft visualization and quantification in allogeneic transplantation mice model, compared with the non-specific radiotracer ^18^F-FDG under using of immunosuppressant. Labelling, binding, and stability studies were performed. BALB/c mice transplanted with C57BL/6 skin grafts, with or without rapamycin treatment (named as allo-treated group or allo-rejection group), were injected with ^131^I-anti-TLR5 mAb, ^18^F-FDG, or mouse isotype ^131^I-IgG, respectively. Whole-body phosphor-autoradiography and *ex vivo* biodistribution studies were obtained. Whole-body phosphor-autoradiography showed ^131^I-anti-TLR5 mAb uptake into organs that were well perfused with blood at 1 hr and showed clear graft images from 12 hrs onwards. The ^131^I-anti-TLR5 mAb had significantly higher graft uptake and target-to-non-target ratio in the allo-treated group, as determined by semi-quantification of phosphor-autoradiography images; these results were consistent with *ex vivo* biodistribution studies. However, high ^18^F-FDG uptake was not observed in the allo-treated group. The highest allograft-skin-to-native-skin ratio (A:N) of ^131^I-anti-TLR5 mAb uptake was significantly higher than the ratio for ^18^F-FDG (7.68 *versus* 1.16, respectively). ^131^I-anti-TLR5 mAb uptake in the grafts significantly correlated with TLR5 expression in the allograft area. The accumulation of ^131^I-IgG was comparable in both groups. We conclude that radiolabelled anti-TLR5 mAb is capable of detecting allograft with high target specificity after treatment with the immunosuppressive drug rapamycin.

## Introduction

Diagnostic and predictive monitoring tools are desperately needed to customize the delivery of immunosuppressive drugs for optimal patient and organ care. Conventional biomarkers in routine clinical use for transplanted kidney evaluation, including measures of allograft function, such as serum creatinine, provide no information about the immune status of the transplant recipient [1]. Although a biopsy provides invaluable information about the local immune response, which tightly related with allograft dysfunction, the histological and immunohistochemical analysis of biopsy sample alone cannot be employed to predict the development of transplantation tolerance. Thus, in diagnostics, entirely image-based and tolerance-related methods would be superior.

There has been a rapid growth of *in vivo* molecular imaging of transplanted organs based on the molecular and immunological features of rejection, such as infiltrating T-lymphocyte metabolic activity [2,3], consecutive cytokine release [4], cell death [5], and graft function [6,7]. None of these measures are specific for grafts, and all are easily impaired by immunosuppressive medications. Moreover, patients administrated with immunosuppressive drugs are prone to autoimmune inflammatory conditions, rendering such non-specific biomarkers even weaker. ^18^F-FDG has been reported to evaluate acute allograft rejection and to monitor treatment efficacy in an animal rejection model, but the ^18^F-FDG signal in the graft disappears after 24 hrs of cyclosporine A (CsA) application [8]. Thus, as a routine biomarker, ^18^F-FDG may not be suitable for allograft detection when clinical immunosuppressant drugs have been used. No study has been performed to address the application of tolerance-related biomarkers in graft imaging. The absence of sufficiently robust biomarkers further complicates the clinical management of allograft recipients; better diagnostic biomarkers could potentially correlate with the state of the graft and could improve outcome.

As one of the Toll-like receptor family members, TLR5 is expressed in the myelomonocytic cell membrane and recognizes bacterial flagellin [9]. High TLR5 expression has been observed in CD4^+^CD25^+^ Treg cells, and such high expression potently increases the suppressive capacity of these cells *via* enhanced Foxp3 expression [10]; activation of TLR5 by flagellin reduces GvHD (graft-*versus*-host disease) following allogeneic haematopoietic stem cell transplantation [11]. We have previously found that recombinant flagellin (rFlic) collaborates to strengthen tolerance induction by immunosuppressive drug rapamycin [12]. Despite its immune regulatory function, the relationship between TLR5 expression and graft tolerance has not been studied.

## Materials and methods

### Ethics statement

The animal protocol was reviewed and approved by the Institutional Animal Care and Use Committee at the School of Medicine, Shandong University.

### Allogeneic skin transplant model

Female C57BL/6 (H-2^b^) and BALB/c mice (H-2^d^) with free access to standard mouse chow and tap water were used (18 ± 2 g). Experiments were performed in accordance with national animal protection guidelines. Transplantation was performed as previously described [13]. Briefly, full-thickness skin grafts from donor C57BL/6 mice were transplanted onto the prepared graft beds on the right shoulders of recipient BALB/c mice. Surgeries were performed under anaesthesia with 0.6% pentobarbital sodium (0.1 ml/10 g bodyweight) intraperitoneally (i.p.) during the entire operation to control analgesia.

The mice were divided into two groups: the allo-treated group (rapamycin, Sigma-Aldrich Trading Co., Ltd, St. Louis, MO, USA, 1.5 mg/kg D i.p., *n* = 40) and the allo-rejection group (equivalent volume of PBS i.p., *n* = 40).

### Radioiodination of anti-TLR5 mAb and control IgG

Sodium iodide [^131^I] (half-life = 8.04 days) was purchased from the China Institute of Atomic Energy (Beijing, China). Radioiodination of mouse anti-TLR5 mAb (100 μg/ml; Santa Cruz Biotechnology, Inc., Dallas, Texas, USA) and mouse isotype IgG (1 mg/ml; Biosynthesis Biotechnology Co., Ltd., Beijing, China) with ^131^I was performed according to the iodogen method, as previously described [14]. Mouse IgG served as a specific control antibody. Radioiodinated anti-TLR5 mAb and IgG were separated from free iodine using size-exclusion columns (PD-10 Sephadex G-25, GE-Healthcare, Diegem, Belgium), and the flowthrough was collected in sequential fractions. The radioactivity and concentration were measured using a gamma counter (Capintec Inc., Ramsey, NJ, USA).

### Quality control of ^131^I-anti-TLR5 mAb and ^131^I-IgG

The radiochemical purity of the radiolabelled antibodies was determined by size-exclusion high-performance liquid chromatography (SE-HPLC) and radio-thin-layer chromatography (Mini-Scan radio-TLC Strip Scanner, Bioscan, Washington, DC, USA).

The HPLC system (Dionex UltiMate 3000, Sunnyvale, California, USA) used consisted of a manual injector with a 20-μl injection loop (7725i injector, Rheodune LLC, Rohnert Park, CA, USA), an HPLC pump, a variable wavelength detector and an in-line radioactivity detector coupled to a multichannel analyser. Chromatograms were analysed using the Chromeleon software (Dionex). A MAbPac™ SEC-1 size-exclusion column (Dionex) was used. The mobile phase consisted of 50 mM sodium phosphate, pH 6.8, and 300 mM NaCl. The flow rate was 0.20 ml/min., and the UV-detector wavelengths were set to 280 nm at 25°C. The retention time of the anti-TLR5 mAb was 10.9 min. Radioactivity was determined by thin-layer (Mini-Scan radio-TLC Strip Scanner; Bioscan) and paper chromatography.

### *In vitro* evaluation of radiolabelled compounds

Radioligand-based binding assays were performed as previously described [15] and were conducted in test tubes. For saturation studies, the reaction mixture contained 200 μl of splenocytes [16] (5 × 10^6^) and 100 μl of ^131^I-anti-TLR5 mAb (diluted in PBS, 0.1–30 nM), with a final volume of 500 μl. Non-specific binding was evaluated by the presence of anti-TLR5 mAb (diluted in PBS, 50 nM–15 μM) in the same tubes. For competitive binding, 0.1 and 1000 nM anti-TLR5 mAb and 13 nM ^131^I-anti-TLR5 mAb were used. The mixture was incubated at 37°C for 45 min. The bound and free radioactive particles were separated by rapid vacuum filtration through Whatman GF/B filters using a cell harvester followed by 3 × 2 ml washes with PBS at room temperature. The radioactivity of the filters containing the bound radioligand was assayed in test tubes using a Wipe Test/Well Counter (Capintec Inc., Ramsey, NJ, USA). The results of the saturation and competition binding experiments were subjected to non-linear regression analysis using GraphPad Prism 5. This analysis was used to calculate the *K*_d_, *K*_i_ and B_max_ values.

The stability of ^131^I-anti-TLR5 mAb and ^131^I-IgG was determined by storing the final product (2 μl) in 400 μl NS (normal saline, 0.9% NaCl solution) and 400 μl mouse serum at 37°C for 96 hrs. Experiments were performed with a radio-TLC Strip Scanner at 0, 12, 24, 48, 72 and 96 hrs after labelling.

### Whole-body phosphor-autoradiography

^18^F-FDG: Five mice from each group were injected intravenously *via* the tail vein with ^18^F-FDG (5.55 MBq in 150 μl of NS) on day 10 after transplantation. Images were acquired at 30, 60 and 90 min. after injection.

^131^I-anti-TLR5 mAb and ^131^I-IgG: Two days before injection of radiotracers, 10% potassium iodide was added to the drinking water to block the thyroid gland. Five mice from each group were injected intravenously *via* the tail vein with a PBS solution containing ^131^I-anti-TLR5 mAb or ^131^I-IgG (150 μl, 0.37 MBq) on day 7 after transplantation. Images were acquired at 1, 12, 24, 48 and 72 hrs after injection.

Dynamic whole-body phosphor-autoradiography was performed with the Cyclone Plus Storage Phosphor System (PerkinElmer, Waltham, MA, USA). During the acquisition, the transplanted mice were anaesthetized with 0.60% pentobarbital sodium (0.1 ml/10 g body wt) i.p. Anaesthetized mice were placed on the storage phosphor screen plate with their backs facing the plate in low light. The plate was exposed to a mouse for 10 min. Following exposure, the plate was immediately covered with an opaque plastic sheet, then transferred to the scanner (Cyclone Plus, PerkinElmer) and scanned.

### Image analysis and quantitative evaluation

To delineate the kidney contours, representative images were chosen at 90 min. of exposure to ^18^F-FDG and 72 hrs of exposure to ^131^I-labelled antibodies. Semi-quantitative results were obtained by manually drawing rectangular regions of interest (ROIs) [5 ROIs per graft (*n* = 5)] in the target area. Digital light units (DLU) per mm^2^ were then calculated using OptiQuant™ image analysis software (PerkinElmer).

### *Ex vivo* biodistribution

For the biodistribution study, 25 mice were injected with ^31^I-anti-TLR5 mAb or ^131^I-IgG (150 μl, 0.37 MBq). Five mice were killed at 1, 12, 24, 48 and 72 hrs after injection. Organs and tissues were excised, rinsed to remove residual blood, and weighed. Samples and primed standards were evaluated for radioactivity in the gamma counter and were corrected for physical decay. Tissue activity is expressed as the per cent injected dose per gram (%ID/g). The target-to-non-target ratio was defined as the allograft-skin-to-native-skin ratio (A:N) and the ratio of the allograft to blood.

### Immunohistochemical staining and analysis

Twenty-five mice from two groups (12 mice of allo-rejection group and 13 mice of allo-treated group) were killed at 72 hrs after ^131^I-anti-TLR5 mAb (150 μl 0.37 MBq) injection. The radiotracer radioactivity (DLU/mm^2^) was obtained by OptiQuant™ image analysis software (PerkinElmer).

Grafts were harvested for immunohistochemical staining with rabbit polyclonal TLR5 antibody (Biosynthesis Biotechnology Co., Ltd.), and DAB chromogen (Biogenics, Napa, CA, USA). Immunohistochemistry was performed with SP-9002 Histostain™ Plus kits (ZSGB-BIO, Beijing, China) according to the manufacturer's protocols. The slides were visualized with an inverted biological microscope (DMIRB; Leica, Wetzlar, Hesse-Darmstadt, Germany) at a magnification of 100×. Corresponding positive areas of slides were analysed by the Image-Pro Plus software version 4.5.0.29 (Media Cybernetics, Bethesda, USA), and stained areas, integral optical density (IOD) were determined. Measurements were performed in five fields per slides. Values after subtracting background density were averaged to give an IOD value. The mean density (IOD/area) was calculated.

### Data analysis

Quantitative data are expressed as means ± SD. Statistically significant differences in ^131^I-anti-TLR5 mAb uptake between allografts and native skin were assessed using Student's *t*-test (unpaired, two-tailed) in the GraphPad Prism 5 software. Pearson's correlation analysis was used to assess the relationship between TLR5 expression and ^131^I-anti-TLR5 mAb uptake using the Prism package. Data regarding the mean density of TLR5 expression in the grafts (IOD/Area) and uptake values from two groups were treated as one entity. *P* values of less than 0.05 were considered to be statistically significant.

## Results

### Radioiodination

Labelling with ^131^I resulted in labelling yields of 96.90% ± 0.60% for ^131^I-anti-TLR5 mAb and 96.62% ± 0.74% for ^131^I-IgG. The specific radioactivities were measured at 458.6 MBq/mg and 407.2 MBq/mg; no impurities were detected. These results demonstrate that ^131^I-anti-TLR5 mAb can be ^131^I-labelled with a high labelling efficiency.

### *In vitro* evaluation of radiolabelled compounds

**Fig. 1:**
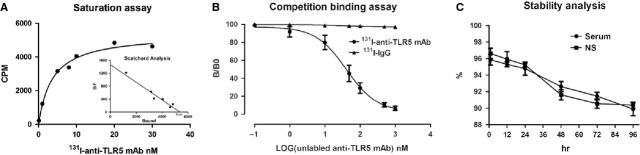
*In vitro* evaluation of radiolabelled compounds. Representative saturation binding and Scatchard plots of ^131^I-anti-TLR5 mAb binding to the membranes of isolated splenocytes from BALB/c mice (**A**). The concentration of the labelled ligand (anti-TLR5 mAb and mouse IgG) was held constant, and increasing concentrations of unlabelled anti-TLR5 mAb were used to compete with the binding (**B**). Stability analysis of ^131^I-anti-TLR5 mAb in the serum or NS within 96 hrs (**C**). NS, normal saline.

As shown in Figure 1, we identified a single-affinity class of sites in splenocyte membranes. A Scatchard plot and computer curve fitting of the saturation data revealed a *K*_d_ value of 3.67 and a *B*_max_ value of 5.39 μM (Fig. 1A). Competitive binding analysis with excess unlabelled anti-TLR5 mAb (>500-fold) could almost completely block the ^131^I-anti-TLR5 mAb (<5%) with a *K*i value of 8.89, comparable with the 3–7% non-specific binding observed for ^131^I-IgG (Fig. 1B). The ^131^I-anti-TLR5 mAb displayed high protein-bound radioactivity (>88%) in both mouse serum and NS after 96 hrs (Fig. 1C). These results ensure adequate TLR5 binding by ^131^I-anti-TLR5 mAb and optimal quantitative studies over 72 hrs.

### Whole-body phosphor-autoradiography

**Fig. 2:**
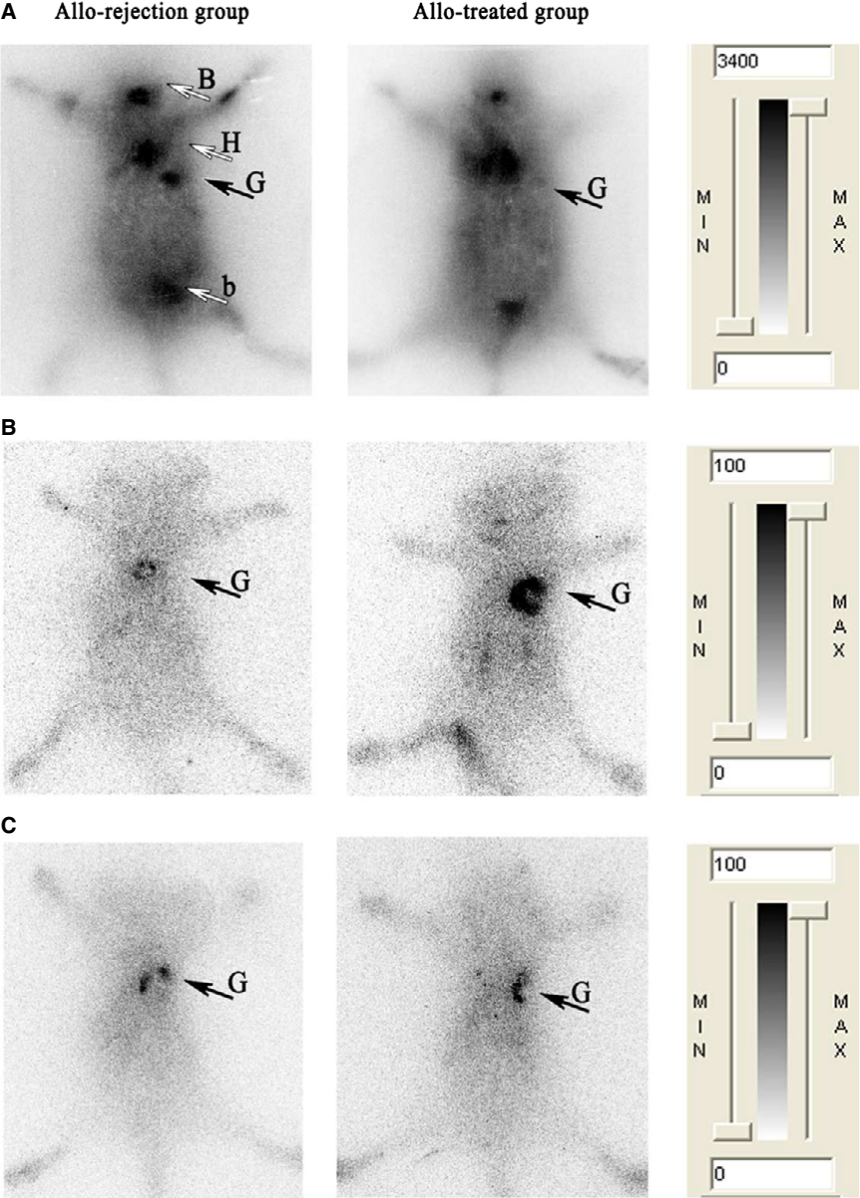
Whole-body phosphor-autoradiography of three radiotracers. Representative images of ^18^F-FDG showed significant accumulation in the allo-rejection graft 90 min. after injection (**A**). Representative images of ^131^I-anti-TLR5 mAb showed significant accumulation in the allo-treated group 72 hrs after injection (**B**). Representative images of ^131^I-IgG showed similar levels of accumulation in both groups (**C**). B, brain; H, heart; G, C57BL/6 skin graft; b, bladder.

**Fig. 3:**
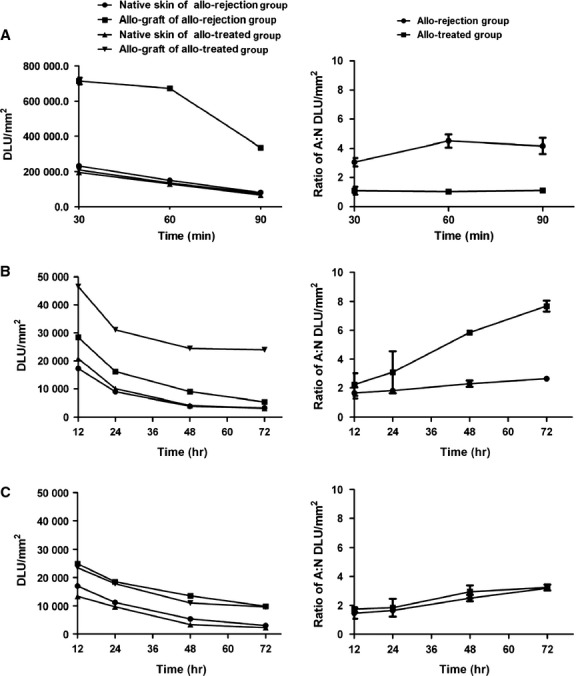
Dynamic time-activity curves (left) and ratio of allograft to native skin (right) of whole-body phosphor-autoradiography images were performed at different time-points using Cyclone Plus Storage Phosphor System (PerkinElmer) after tail vein injection three radiotracers (**A**, ^18^F-FDG; **B**, ^131^I-anti TLR5 mAb; **C**, ^131^I-IgG). Regions of interest (ROIs) in the target area were obtained from the images.

**Fig. 4:**
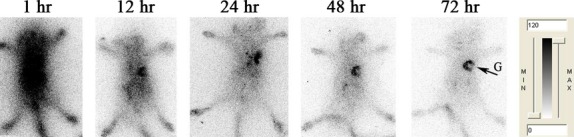
Dynamic whole-body phosphor-autoradiography of TLR5 expression in allo-treated grafts with ^131^I-anti-TLR5 mAb (150 μl, 0.37 MBq) was performed at 1, 12, 24, 48 and 72 hrs after injection. Solid arrow = C57BL/6 skin graft.

Whole-body phosphor-autoradiography enabled excellent graft visualization only in the allo-rejection group compared with the allo-tolerance group 90 min. after ^18^F-FDG injection (Fig. 2A). Radioactivity (DLU/mm^2^) of allograft area was from 33,6314.6 ± 23,998.4 to 74,949.6 ± 4370.1 (Fig. 3A, left). The highest A: N of ^18^F-FDG in all-treated group was 1.16 ± 0.01 compared to 4.50 ± 0.34 in allo-rejection group (Fig. 3A, right). Organs with high uptake included the brain, heart and bladder in both groups. Obviously, rapamycin application impaired ^18^F-FDG uptake in the graft area. In contrast, the ^131^I-anti-TLR5 mAb was taken up in well-perfused organs and in the plasma at 1 hr after injection in the allo-treated group, which declined at later time-points. Increasing the allograft-to-blood ratio resulted in clear graft imaging of the ^131^I-anti-TLR5 mAb from 12 hrs onwards after injection (Fig. 4). Higher ^131^I-anti-TLR5 mAb uptake occurred in the allo-treated group compared to the allo-rejection group, as assessed by whole-body phosphor-autoradiography images at 72 hrs after ^131^I-anti-TLR5 mAb injection (Fig. 2B). Radioactivity (DLU/mm^2^) of allograft area was from 24,021.7 ± 261.2 to 5441.4 ± 85.2 (Fig. 3B, left). The highest A: N of ^131^I-anti-TLR5 mAb in all-treated group was 7.68 ± 0.38 compared to 2.64 ± 0.08 in allo-rejection group (Fig. 3B, right).

IgG accumulated non-specifically in the local inflammatory area *via* the Fc portion binding to the Fc receptors of immune effector cells [17,18]. Because anti-TLR5 mAb belongs to the mouse IgG_2a_ subtype, we labelled mouse isotype IgG with ^131^I to illustrate the non-specific binding. The phosphor-autoradiography experiments showed an equal organ density for both groups at 72 hrs after ^131^I-IgG injection (Fig. 2C); ^131^I-IgG uptake and the A: N ratio in the allograft area were much lower than ^131^I-anti-TLR5 mAb uptake in the allo-treated group (Fig. 3C), supporting the conclusion that the uptake of ^131^I-anti-TLR5 mAb was specific to the allo-treated grafts.

### *Ex vivo* biodistribution

To validate the imaging studies and to further quantify the ^131^I-anti-TLR5 mAb uptake, biodistribution studies were performed at 1, 12, 24, 48 and 72 hrs in the allo-treated group (Table 1). The biodistribution studies revealed that the allografts had the highest uptake at all time-points. Other tissues with higher uptake were the liver, kidney and lung. All other tissues had a low %ID/g, in agreement with the imaging data. The allograft-to-blood ratios supported the best contrast observed at 72 hrs after injection.

**Table 1:** Biodistribution of ^131^I-anti-TLR5 mAb in the allo-treated group

	Time (h)				
	1	12	24	48	72
Blood	13.17 ± 1.75	7.10 ± 0.32	3.59 ± 0.88	1.34 ± 0.11	0.66 ± 0.02
Liver	12.89 ± 0.81	5.53 ± 0.49	2.13 ± 0.74	0.87 ± 0.07	0.64 ± 0.09
Kidney	17.50 ± 1.70	8.26 ± 1.36	3.35 ± 0.69	2.46 ± 0.34	0.93 ± 0.11
Spleen	12.40 ± 0.54	4.13 ± 0.74	1.92 ± 0.09	1.38 ± 0.04	0.64 ± 0.02
Stomach	5.43 ± 0.56	3.83 ± 0.96	1.80 ± 0.23	0.63 ± 0.16	0.44 ± 0.12
Bone	2.54 ± 0.35	2.12 ± 0.95	1.95 ± 0.35	0.60 ± 0.18	0.24 ± 0.06
Muscle	2.51 ± 0.76	1.36 ± 0.02	0.94 ± 0.66	0.35 ± 0.19	0.24 ± 0.04
Heart	2.98 ± 0.39	2.28 ± 0.0	1.99 ± 0.08	0.71 ± 0.27	0.27 ± 0.13
Lung	11.12 ± 0.22	7.27 ± 0.71	4.79 ± 0.16	2.27 ± 0.43	0.87 ± 0.15
Thyroid	7.31 ± 0.63	3.17 ± 0.52	1.89 ± 0.94	0.91 ± 0.20	0.36 ± 0.05
Native Skin	5.05 ± 0.66	4.17 ± 0.24	2.35 ± 0.55	0.82 ± 0.13	0.29 ± 0.02
Allograft	12.05 ± 1.86*	10.08 ± 0.78*	7.47 ± 1.46*	3.17 ± 0.06*	1.95 ± 0.38*
Allograft-to-blood ratio	0.91 ± 0.14	1.42 ± 0.08	2.08 ± 0.05	2.36 ± 0.11	2.95 ± 0.12

* *P* < 0.0001 compared to the native skin of the opposite side in the same mouse.

Data are presented as means %ID/g ± SD of 5 animals.

### Correlation between TLR5 expression and ^131^I-anti-TLR5 mAb uptake

**Fig. 5:**
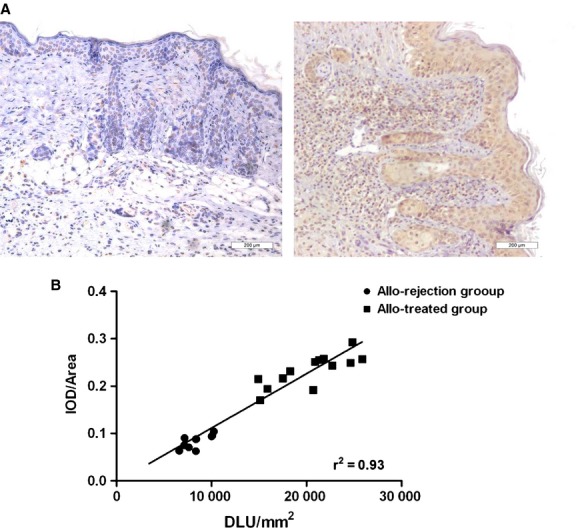
TLR5 immunohistochemical staining and correlation analysis with ^131^I-anti-TLR5 mAb uptake. Representative microscopy images of 10-μm-thick sections stained for TLR5 at 100× magnification from the allo-rejection (left) and allo-treated (right) group (**A**). There was a significant correlation between TLR5 expression and ^131^I-anti-TLR5 mAb uptake (*r*^2^ = 0.93, *n* = 25, *P* < 0.0001). Graft data from two groups were considered as an entity (**B**).

Representative images from the allo-rejection and allo-treated groups stained with TLR5 antibody are shown in Figure 5A. The allo-treated group had a clearly higher TLR5 expression. ^131^I-anti-TLR5 mAb uptake in the grafts from the biodistribution study displayed a significant correlation with the percentage of TLR5 expression (*r*^2^ = 0.93, *P* < 0.0001, *n* = 25, Fig. 5B).

## Discussion

^18^F-FDG is a useful routine biomarker to quantify acute organ rejection and is taken up by activated immune cells because of metabolism of glucose [19–21]. Immunosuppression significantly reduces overall sequestration of glucose tracers, leading to a decrease in ^18^F-FDG uptake and causing images of the grafts to vanish [8,21]. As an mTOR inhibitor, rapamycin can reduce FDG and fluorothymidine uptake in tumour xenografts derived from sensitive tumour cell lines [22,23]. Recipients with ongoing acute lung allograft rejection have dramatically reduced ^18^F-FDG uptake in their grafts after immunosuppression, no matter whether a low or high dose of CsA is used [3]. Our data suggest that immune cells are sensitive to rapamycin; therefore, ^18^F-FDG is not suitable for monitoring graft survival following immunosuppressant drug application.

As the induction and maintenance of donor-specific tolerance is a central aim in solid organ transplantation, a reliably biomarker is essential for clinicians to identify and monitor tolerance accurately. CD4^+^CD25^+^Foxp3^+^Treg is the first choice which associates with allograft tolerance induction. Although it has been suggested by a number of studies reporting an increased circulating proportion of Treg in operationally tolerant transplant recipients [24,25], owing to the little portion of blood cells, the expression of Treg markers were not sufficient to be detected by present imaging methods.

Toll-like receptors play prominent roles in the inflammatory response against pathogen infection by recognizing conserved pathogen-associated molecular patterns [26]. Recent studies suggest that TLR5, the only TLR member that can recognize a protein bacterial product (flagellin), is the most potent immunomodulatory reagent. In the mouse model of *Pseudomonas aeruginosa* infection-induced keratitis, subconjunctival or intraperitoneal injection of flagellin 24 hrs before *P. aeruginosa* infection can significantly improve the prognosis of the disease, protect the structural integrity and transparency of the cornea and reduce polymorphic nuclear leucocyte infiltration and pro-inflammatory cytokine production [27]. Furthermore, TLR5 knockout mice are prone to spontaneous colitis; in contrast, TLR5 overactivation can negatively regulate some inflammatory bowel diseases, such as Crohn's disease and ulcerative colitis [28]. Our prior laboratory research found that use of an immunosuppressive drug (rapamycin) *in vivo* promoted the expression of TLR5 and Foxp3 in a mouse allogeneic transplantation model [29]. Therefore, TLR5 expression may be relevant to rapamycin-induced graft tolerance and Treg may be a part of the source of TLR5-expressing cells.

In the present study, ^131^I labelling of anti-TLR5 mAb resulted in a high labelling efficiency and adequate preservation of TLR5 binding properties. Allo-treated graft uptake of ^131^I-anti-TLR5 mAb was significantly higher compared to both ^18^F-FDG and control ^131^I-IgG. Allo-treated graft uptake of ^131^I-anti-TLR5 mAb was already high 1 hr after injection and increased over time, with clear graft visualization from 12 hrs onwards after injection. These results demonstrate that TLR5 may be a potential new biomarker for the non-invasive imaging of rapamycin-treated allogeneic grafts, and radiolabelled anti-TLR5 mAb can be used as a tracer targeting TLR5 expression. As a control, we used ^131^I-labelled mouse isotype IgG to provide evidence that the ^131^I-anti-TLR5 mAb uptake is because of specific targeting within the graft and is not caused by blood perfusion and non-specific binding within the graft. These results were also substantiated by whole-body phosphor-autoradiography and biodistribution studies. To obtain more detailed information with regard to the localization of ^131^I-anti-TLR5 mAb accumulation in the allograft, we performed antimouse TLR5 staining in the allo-treated and allo-rejection groups. A significant correlation between ^131^I-anti-TLR5 mAb uptake and TLR5 expression was observed. These results suggest that the ^131^I-anti-TLR5 mAb binds primarily to TLR5 present in the grafts.

Antibodies that exceed the molecular weight cut-off of the renal glomerular filtration barrier have long circulation times. The half-life of ^131^I was 8.04 days, matching the pharmacokinetics of IgG_2a_ (half-life = 6–8 days) [30]. The dynamic whole-body phosphor-autoradiography showed good characteristics for antibody imaging with a clear background from 12 hrs after injection.

## Conclusion

We investigated a radiolabelled version of ^131^I-anti TLR5 mAb as a specific imaging agent for TLR5 expression. The specificity, target selectivity and graft-to-non-graft ratio observed suggest that ^131^I-anti TLR5 mAb, compared with the non-specific radioactive indicator ^18^F-FDG, is a superior agent for nuclear imaging of rapamycin-treated allogeneic graft and can delineated graded levels of TLR5 expression in graft. We address the possibility of TLR5 as a new allograft biomarker in non-invasive molecular imaging.

## References

[1] Cravedi P, Mannon RB (2009). Noninvasive methods to assess the risk of kidney transplant rejection. Expert Rev Clin Immunol.

[2] Grabner A, Kentrup D, Edemir B (2013). PET with 18F-FDG–labeled T lymphocytes for diagnosis of acute rat renal allograft rejection. J Nucl Med.

[3] Chen DL, Wang X, Yamamoto S (2013). Increased T cell glucose uptake reflects acute rejection in lung grafts. Am J Transplant.

[4] Fischereder M, Schroppel B (2008). The role of chemokines in acute renal allograft rejection and chronic allograft injury. Front Biosci.

[5] Narula J, Acio ER, Narula N (2001). Annexin-V imaging for noninvasive detection of cardiac allograft rejection. Nat Med.

[6] de Graaf W, Bennink RJ, Veteläinen R (2010). Nuclear imaging techniques for the assessment of hepatic function in liver surgery and transplantation. J Nucl Med.

[7] Freeman AM, Georgakis A, Kochar M (2009). Increased thallium-201 accumulation in the transplanted lung. Clin Nucl Med.

[8] Reuter S, Schnöckel U, Edemir B (2010). Potential of noninvasive serial assessment of acute renal allograft rejection by 18F-FDG PET to monitor treatment efficiency. J Nucl Med.

[9] Hayashi F, Smith KD, Ozinsky A (2001). The innate immune response to bacterial flagellin is mediated by Toll-like receptor 5. Nature.

[10] Crellin NK, Garcia RV, Hadisfar O (2005). Human CD4^+^ T cells express TLR5 and its ligand flagellin enhances the suppressive capacity and expression of FOXP3 in CD4^+^ CD25^+^ T regulatory cells. J Immunol.

[11] Hossain MS, Jaye DL, Pollack BP (2011). Flagellin, a TLR5 agonist, reduces graft-*versus*-host disease in allogeneic hematopoietic stem cell transplantation recipients while enhancing antiviral immunity. J Immunol.

[12] Hao J, Zhang C, Liang T (2013). rFliC prolongs allograft survival in association with the activation of recipient Tregs in a TLR5-dependent manner. Cell Mol Immunol.

[13] Liang T, Zhang C, Song J (2011). Evaluation of< sup> 131</sup> I-anti-MIF mAb as a reporter for allograft rejection. Clin Immunol.

[14] Zhang C, Hou G, Liang T (2009). A prospective study of macrophage migration inhibitory factor as a marker of inflammatory detection. J Cell Mol Med.

[15] Wu C, Wei J, Tian D (2008). Molecular probes for imaging myelinated white matter in CNS. J Med Chem.

[16] Fantini MC, Dominitzki S, Rizzo A (2007). *In vitro* generation of CD4^+^ CD25^+^ regulatory cells from murine naive T cells. Nat Protoc.

[17] Kiekens RCM, Thepen T, Bihari IC (2000). Expression of Fc receptors for IgG during acute and chronic cutaneous inflammation in atopic dermatitis. Br J Dermatol.

[18] Rubin RH, Fischman AJ, Callahan RJ (1989). ^111^In-labeled nonspecific immunoglobulin scanning in the detection of focal infection. N Engl J Med.

[19] Hoff SJ, Stewart JR, Frist WH (1993). Noninvasive detection of acute rejection in a new experimental model of heart transplantation. Ann Thorac Surg.

[20] Jacobs SR, Herman CE, MacIver NJ (2008). Glucose uptake is limiting in T cell activation and requires CD28-mediated Akt-dependent and independent pathways. J Immunol.

[21] Tsuji AB, Morita M, Li XK (2009). 18F-FDG PET for semiquantitative evaluation of acute allograft rejection and immunosuppressive therapy efficacy in rat models of liver transplantation. J Nucl Med.

[22] Cejka D, Kuntner C, Preusser M (2009). FDG uptake is a surrogate marker for defining the optimal biological dose of the mTOR inhibitor Rapamycin *in vivo*. Br J Cancer.

[23] Honer M, Ebenhan T, Allegrini PR (2010). Anti-angiogenic/vascular effects of the mTOR inhibitor Rapamycin are not detectable by FDG/FLT-PET. Transl Oncol.

[24] Li Y, Koshiba T, Yoshizawa A (2004). Analyses of peripheral blood mononuclear cells in operational tolerance after pediatric living donor liver transplantation. Am J Transplant.

[25] Pons JA, Revilla-Nuin B, Baroja-Mazo A (2008). FoxP3 in peripheral blood is associated with operational tolerance in liver transplant patients during immunosuppression withdrawal. Transplantation.

[26] Kutikhin AG (2011). Association of polymorphisms in TLR genesand in genes of the Tolllike receptor signaling pathway with cancer risk. Hum Immunol.

[27] Kumar A, Hazlett LD, Yu FS (2008). Flagellin suppresses the inflammatory response and enhances bacterial clearance in a murine model of *Pseudomonas aeruginosa* keratitis. Infect Immun.

[28] Himmel ME, Hardenberg G, Piccirillo CA (2008). The role of T-regulatory cells and Toll-like receptors in the pathogenesis of human inflammatory bowel disease. Immunology.

[29] Chen FQ, Qu LL, Zhang C (2009). [Effect of Rapamycin and Cyclosporin A on the expression of TLR5 and Foxp3 in allorejection]. Xi Bao Yu Fen Zi Mian Yi Xue Za Zhi.

[30] Vieira P, Rajewsky K (1988). The half-lives of serum immunoglobulins in adult mice. Eur J Immunol.

